# Impact of Herbivore Identity on Algal Succession and Coral Growth on a Caribbean Reef

**DOI:** 10.1371/journal.pone.0008963

**Published:** 2010-01-29

**Authors:** Deron E. Burkepile, Mark E. Hay

**Affiliations:** 1 School of Biology, Georgia Institute of Technology, Atlanta, Georgia, United States of America; 2 Department of Biological Sciences, Marine Sciences Program, Florida International University, North Miami, Florida, United States of America; Northeastern University, United States of America

## Abstract

**Background:**

Herbivory is an important top-down force on coral reefs that regulates macroalgal abundance, mediates competitive interactions between macroalgae and corals, and provides resilience following disturbances such as hurricanes and coral bleaching. However, reductions in herbivore diversity and abundance via disease or over-fishing may harm corals directly and may indirectly increase coral susceptibility to other disturbances.

**Methodology and Principal Findings:**

In two experiments over two years, we enclosed equivalent densities and masses of either single-species or mixed-species of herbivorous fishes in replicate, 4 m^2^ cages at a depth of 17 m on a reef in the Florida Keys, USA to evaluate the effects of herbivore identity and species richness on colonization and development of macroalgal communities and the cascading effects of algae on coral growth. In Year 1, we used the redband parrotfish (*Sparisoma aurofrenatum*) and the ocean surgeonfish (*Acanthurus bahianus*); in Year 2, we used the redband parrotfish and the princess parrotfish (*Scarus taeniopterus*). On new substrates, rapid grazing by ocean surgeonfish and princess parrotfish kept communities in an early successional stage dominated by short, filamentous algae and crustose coralline algae that did not suppress coral growth. In contrast, feeding by redband parrotfish allowed an accumulation of tall filaments and later successional macroalgae that suppressed coral growth. These patterns contrast with patterns from established communities not undergoing primary succession; on established substrates redband parrotfish significantly reduced upright macroalgal cover while ocean surgeonfish and princess parrotfish allowed significant increases in late successional macroalgae.

**Significance:**

This study further highlights the importance of biodiversity in affecting ecosystem function in that different species of herbivorous fishes had very different impacts on reef communities depending on the developmental stage of the community. The species-specific effects of herbivorous fishes suggest that a species-rich herbivore fauna can be critical in providing the resilience that reefs need for recovery from common disturbances such as coral bleaching and storm damage.

## Introduction

On coral reefs large herbivores such as fishes and sea urchins can remove >90% of the daily seaweed production and maintain a “grazing lawn” of small, highly productive, algal turfs that supports a large portion of the secondary production on reefs [1], [2], [3]. However, when grazing rates are lowered due to herbivore removal or the opening of new substrate following coral mortality, macroalgae often become abundant [2], [4], [5], [6], [7]. Abundant macroalgae can lower the growth, fecundity, and survivorship of established corals [4], [5], [8], [9], suppress the recruitment and survival of juvenile corals [10], [11], and increase the prevalence of coral disease [12]. Thus, high grazing rates are important for minimizing the negative impacts of macroalgae on coral recruitment, growth, and survivorship and may help facilitate recovery of coral populations in areas where corals have declined [5], [13], [14].

In response to the biodiversity crisis and the need to understand the links between biodiversity and ecosystem function, increasing importance is now placed on identifying the roles of particular herbivore species and the role of herbivore richness and diversity in driving the community dynamics on reefs [15], [16], [17], [18]. Theory suggests that herbivore diversity should benefit reefs because different herbivores have different attack strategies, decreasing the probability that any given macroalga will be well defended against all herbivores [19], [20]. Thus, an increased diversity of herbivores should more efficiently suppress macroalgae and produce positive indirect effects on coral settlement, growth, and reproduction. Herbivore diversity could be especially critical on Caribbean coral reefs because these reefs are species poor compared to reefs in many other regions [21] and because herbivorous fishes are heavily exploited in many areas of the Caribbean [22]. Thus, it may be especially critical to understand how changing the abundance and diversity of herbivorous fishes will impact reef organization and function. Coral cover in the Caribbean has decreased on average by 80% in recent decades [23] due to a number of stressors such outbreaks of coral disease, coral bleaching, disturbances such as hurricanes, eutrophication, and alterations to trophic interactions [24], [25], [26]. These effects have substantially altered many reefs in the Caribbean making investigations into the processes and mechanisms that promote coral resilience increasingly important.

Observational studies of herbivorous fishes in the Caribbean show important among-species differences in diet selection, bioerosion rates, and foraging behavior [27], [28], [29], [30], [31] suggesting that different species may produce different direct and indirect effects on reef community structure. Although observational studies of herbivorous fishes are important for documenting feeding behavior and patterns, they cannot assess unambiguously the complex, direct and indirect effects of herbivore identity and richness on algal communities and coral fitness. These direct and indirect effects can be evaluated only by using controlled experimentation [32], albeit with limitations. Here we report the results of two experiments conducted over two years that assess how herbivore identity and species richness affected the recruitment and primary succession of algal communities and the effects of this on coral growth. We enclosed equivalent densities and biomasses of single-herbivore versus mixed-herbivore groups of fishes in large, replicate cages on a reef in the Florida Keys, USA and monitored algal community development and coral growth on new substrates (cinderblocks) over 7–10 months each year. In Year 1, we used the redband parrotfish (*Sparisoma aurofrenatum*) and the ocean surgeonfish (*Acanthurus bahianus*) to generate the treatments; in Year 2, we used the redband parrotfish and the princess parrotfish (*Scarus taeniopterus*). These species represent the three dominant genera of herbivorous fishes on Caribbean reefs, are among the more common species [33], and have a range of adaptations for herbivory [34]. We show significant effects of herbivore identity in controlling the trajectory of primary succession and coral growth but minimal effects of herbivore richness. The lack of a richness effect contrasts significantly with previously documented effects of these same fishes in these same cages on established algal communities where both herbivore identity and herbivore richness effects were strong [16].

## Results

### Effects of Herbivore Richness and Identity on the Algal Community

**Figure 1:**
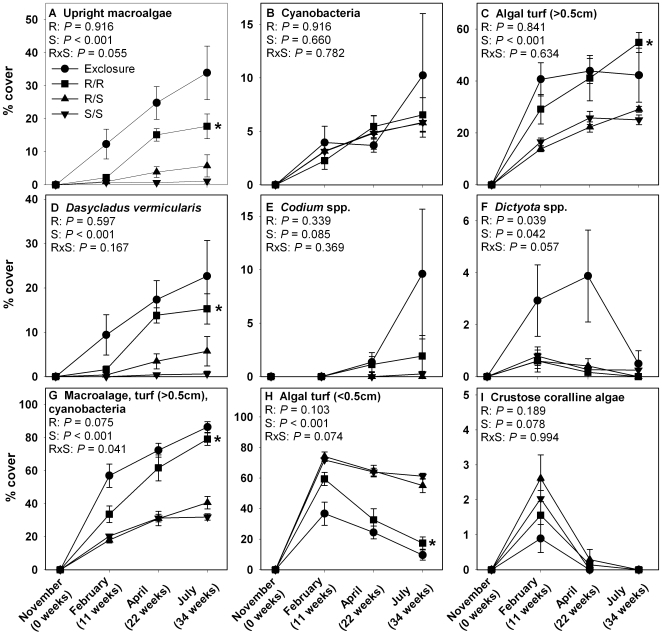
Percent cover (mean ± SE) of common algal types over time during Year 1. R  =  redband parrotfish and S  =  ocean surgeonfish. Statistics are from repeated measures, two-factor ANOVA. * indicates a single-herbivore treatment that differs from the mixed-herbivore treatment as determined via resampling statistics. *n* = 8 for Exclosure, *n* = 6 for R/R, *n* = 6 for R/S, and *n* = 8 for S/S. Note different Y-axes.

**Figure 2:**
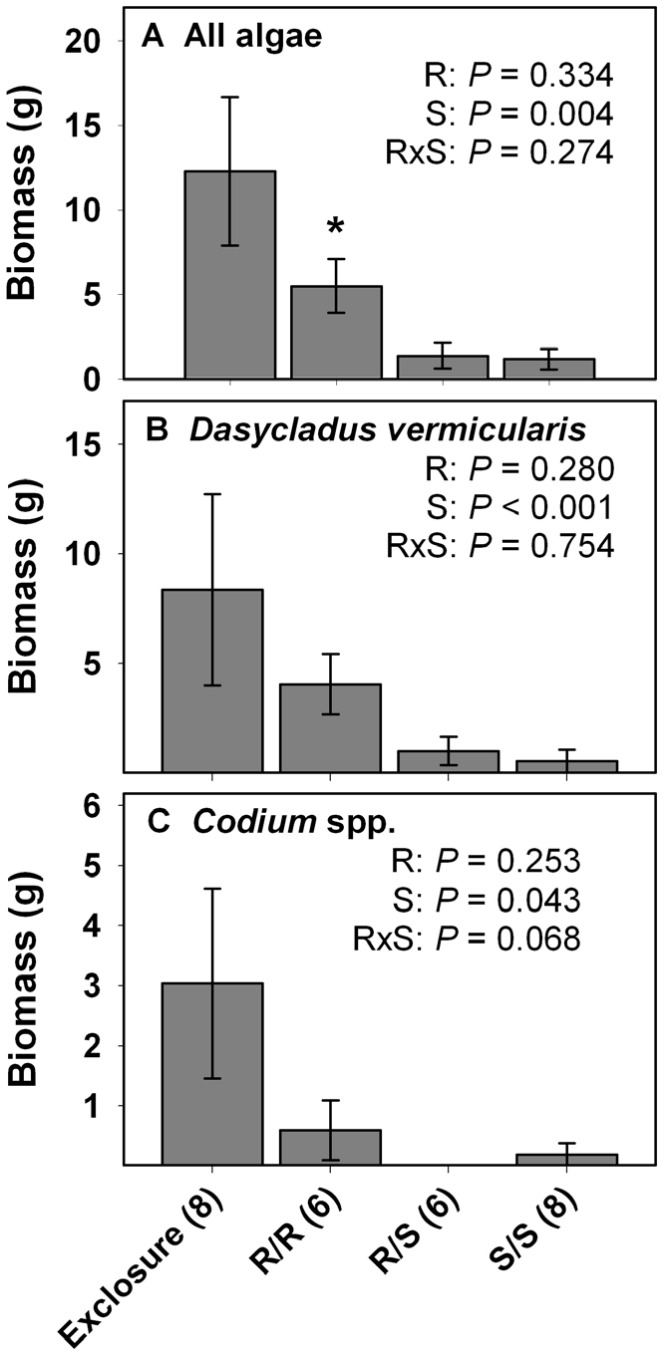
Biomass (mean ± SE) of algae at the end of Year 1. Data are for (A) all algae, (B) *Dasycladus vermicularis*, and (C) *Codium* spp. Statistics are from two-factor ANOVA. * indicates that single- vs. mixed-herbivore treatments differ as determined via resampling statistics. Sample size is in parenthesis next to each treatment.

In Year 1, ocean surgeonfish suppressed colonization of upright macroalgae, taller algal turf, the composite group of upright macroalgae, cyanobacteria, and taller algal turf, and the common macrophytes *Dasycladus vermicularis* and *Dictyota* spp., while enhancing short algal turf (<0.5 cm) (Fig. 1, Table S1). Surgeonfish also suppressed the accumulation of overall algal biomass as well as biomass of *Dasycladus vermicularis* and *Codium* spp. (Fig. 2). In contrast to surgeonfish, redband parrotfish had no significant effects on the cover or biomass of any algal group except for *Dictyota* spp. Algal abundances for the redband-only treatments were similar to the herbivore exclosures (Fig. 1 & 2, Table S1). Other common macrophytes on Caribbean reefs, such as *Lobophora variegata* and *Halimeda* spp., were not significant parts of the algal community on cinderblocks and represented <1% of the community in all treatments. The trajectories of change for most algal groups appeared consistent throughout the experiment with little apparent seasonality (Fig. 1).

**Figure 3:**
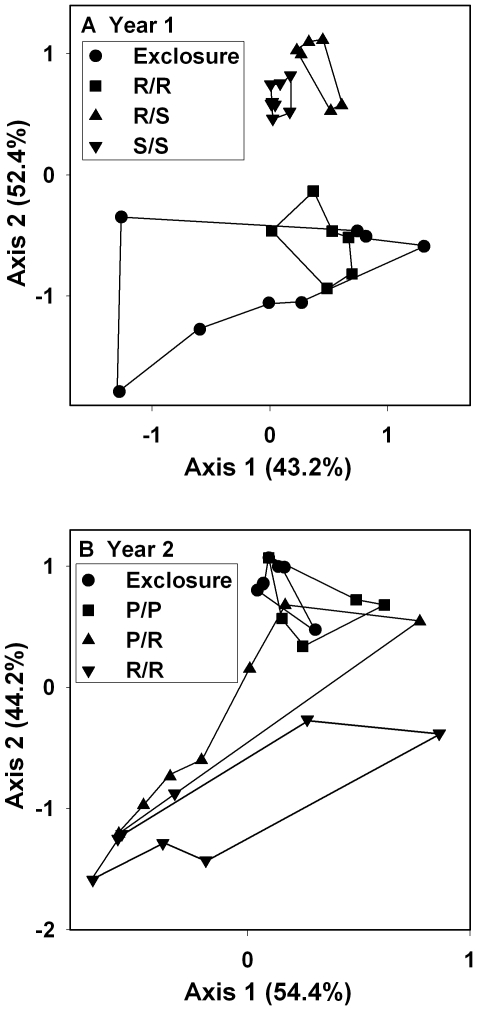
Plot of axes from nonmetric multidimensional scaling (NMDS) describing the similarity of the algal communities among the treatments. Data are for (A) Year 1 and (B) Year 2. Values within parentheses show % of variance explained by each axis. Symbols are as in Fig. 1. Lines connect replicates within treatment.

Resampling statistics showed no significant effects of herbivore richness on individual algal groups (Fig. 1 & 2). The decrease in replication in several treatments following intrusion by moral eels (see Materials and Methods) may have decreased our statistical power making it more difficult to detect differences between some mixed-species and single-species treatments. However, this did not affect the detection of herbivore richness effects on established substrates [16], and did not appear to be a problem here because the mixed-species treatment was usually intermediate between the two single-species treatments rather then either higher or lower than both single-species treatments, the sign of a richness effect. Nonmetric multidimensional scaling (NMDS) analysis of the algal communities showed that the surgeonfish-only and mixed-herbivore treatments clustered closely together in axis space (Fig. 3A). The redband-only treatment clustered in distinctly different axis space, being most similar to the exclosure treatments. In feeding assays, neither redband parrotfish nor ocean surgeonfish consumed detectable amounts of adult *Dasycladus vermicularis* (2.1±2.1% removed, *P*>0.5, *df* = 5; 2.3±1.6% removed, *P*>0.5, *df* = 7, respectively).

**Figure 4:**
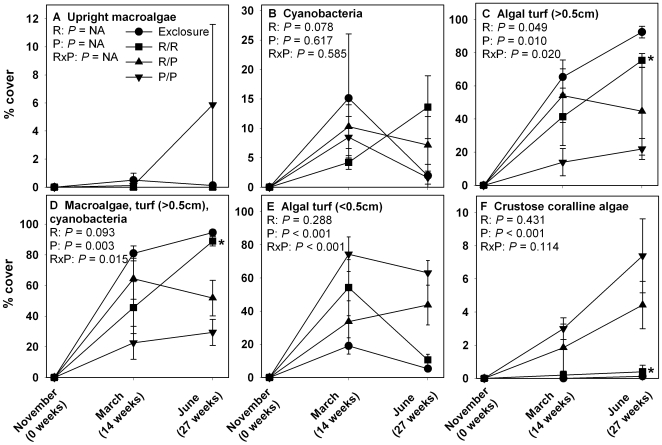
Percent cover (mean ± SE) of common algal types over time during Year 2. R  =  redband parrotfish and P  =  princess parrotfish. Statistics as in Fig. 1, except the general absence of upright macroalgae prevented meaningful statistical analysis for Fig. 4A. *n* = 8 for Exclosure, *n* = 8 for P/P, *n* = 7 for P/R, and *n* = 5 for R/R.

In Year 2, upright macroalgae were generally low in abundance (Fig. 4A), but other algal groups showed significant patterns. Princess parrotfish suppressed accumulation of both taller algal turfs and the combined cover of upright macroalgae, cyanobacteria, and taller algal turf, while enhancing shorter algal turfs and crustose coralline algae (Fig. 4, Table S2). As in Year 1, algal cover in the redband-only treatments was similar to that of the herbivore exclosures for most algal groups with redband parrotfish facilitating accumulation of taller algal turfs (Fig. 4). Resampling statistics showed no effects of herbivore richness for individual algal groups. NMDS showed that the herbivore exclosure and redband-only treatments had similar community structure and clustered closely in axis space (Fig. 3B). The princess-only treatment clustered differently than these two treatments while the mixed-herbivore treatment showed large variation in community structure.

### Effects of Treatments on Coral Growth

**Figure 5:**
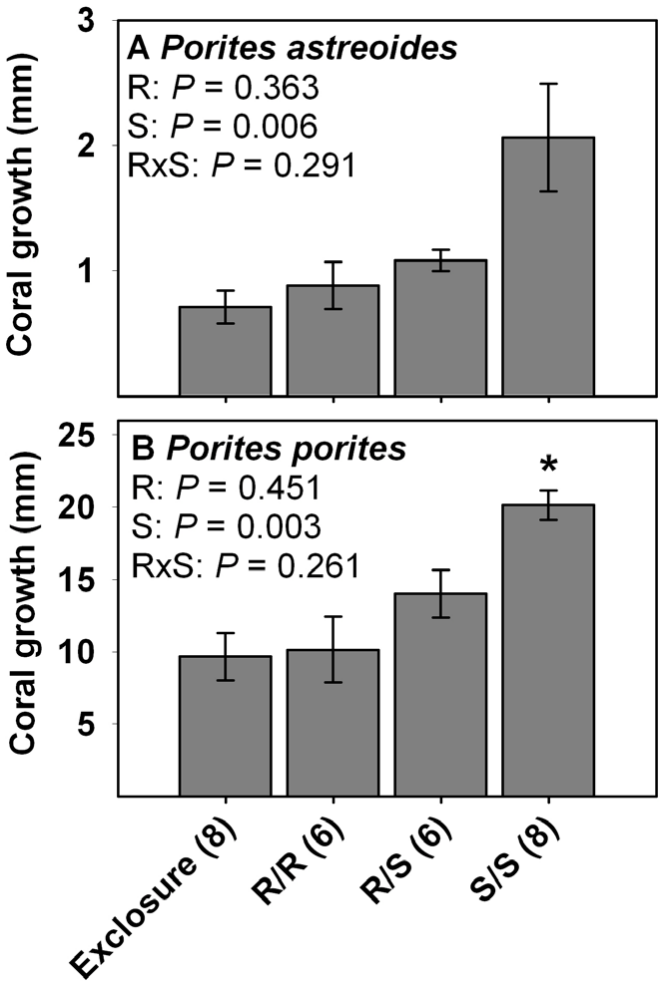
Skeletal growth (mean ± SE) of corals at the end of Year 1. Data are for (A) Porites astreoides and (B) Porites porites. R  =  redband parrotfish and S  =  ocean surgeonfish. Statistics are from two-factor ANOVA. * indicates a single-herbivore treatment that differs from the mixed-herbivore treatment as determined via resampling statistics. Sample size is in parenthesis next to each treatment.

Ocean surgeonfish enhanced the growth of *Porites astreoides* and *Porites porites* by 2-3X, while redband parrotfish had no effects on either coral (Fig. 5). There was no effect of herbivore richness for the growth of either coral species. Although some *Sparisoma* spp. feed directly on corals [35], [36], we detected no grazing scars on either coral species while sectioning them to measure growth, suggesting negligible influence of predation on net growth in the enclosures housing redband parrotfish. Across treatments, linear regression showed significant negative correlations between total algal cover and growth of *P. astreoides* (slope = −0.017, *r* ^2^ = 0.216, *P* = 0.007) and *P. porites* (slope = −0.14, *r* ^2^ = 0.332, *P* = 0.001). These results are consistent with direct competition with algae leading to decreased coral growth rates in those treatments where algae became abundant.

## Discussion

Herbivores often affect succession in marine ecosystems [19], but these impacts may depend on the herbivore species present and the developmental stage of the plant community [37], [38]. For communities undergoing primary succession in our experiments (similar to new substrates created following coral death from bleaching, disease, or storm damage), we showed strong species-specific effects of herbivores with both ocean surgeonfish and princess parrotfish limiting the abundance of late-successional algae and facilitating early successional species such as filamentous algal turfs. These herbivores also facilitated crustose coralline algae, which are commonly associated with increased coral recruitment [39]. In contrast, redband parrotfish had minimal impact on primary succession; they facilitated macrophytes that led to algal communities similar to those in herbivore exclosures. As a result grazing by surgeonfish enhanced coral growth, while coral growth in redband parrotfish treatments was similar to herbivore exclosures. These patterns differ markedly from those for the established benthic community where we also saw strong species-specific effects but in opposite directions. Ocean surgeonfish and princess parrotfish suppressed individual macrophyte species but not overall macroalgal cover while in general facilitating later successional macroalgae on established communities [16]. Redband parrotfish, however, strongly reduced cover of upright macroalgae on established benthic communities. Further, herbivore richness effects were strong on established communities due to complementarity feeding among fish species; this complementary feeding not only impacted seaweeds, but also enhanced coral survivorship and growth [16]. We were unable to detect any such consumer-richness effects on primary succession in this study, suggesting that individual species rather than consumer richness determined the trajectory of algal colonization on new substrates.

One potential concern with our experimental design is that herbivores may change their feeding behavior and diet choice as a consequence of being enclosed. However, enclosed fishes: (1) did not feed faster or slower than free-ranging conspecifics, (2) did not differ in mass/length relationships at the end of the 10 month experiment, and (3) when enclosed and free-ranging conspecifics were offered choices among macrophytes, preferences were similar [16]. We did detect among-species differences in feeding rates within our cages, with princess parrotfishes and ocean surgeonfishes typically biting the benthos >2X as often as redband parrotfishes [16], but these differences reflect natural feeding patterns that also exist for free-ranging fishes. Additionally, had the enclosed fishes expanded their feeding preferences in response to being caged, these changes would have biased our experiments against seeing the strong interspecific differences in effects on primary succession that we documented. Thus, the significant differences that exist among treatments are likely the result of natural differences in the feeding by these fishes rather than an artifact of enclosure.

The impact of consumer richness on communities often varies with the diversity of the prey community [40], [41] which may explain the lack of herbivore richness effects on primary substrates. As compared to the more diverse, established reef communities, the algal communities on experimental blocks were less diverse and more homogeneous; this may have minimized the importance of complementary feeding among herbivore species over the course of our experiment. Had the areas undergoing primary succession been larger and more heterogeneous, then individual herbivore species might have been unable to consistently keep the areas in early successional turfs and the effects of consumer richness documented for established communities on natural substrates [16] may have become more important (see also 24). Additionally, species richness effects can strengthen through time [42], an our experiments lasted only 7–10 months. However, we saw significant herbivore richness effects on established benthic communities during these experiments and over this same time interval [16]. Additionally, depth could be an important factor changing biodiversity-ecosystem function relationships on coral reefs as a variety of physical and biological factors change with depth [43]. For example, algae on shallow coral reefs are more productive than on mid-depth reefs, such as our field site, due to higher light levels and greater flow rates [44]. However, this increased productivity is often compensated for by higher herbivore density and grazing rates on shallow reefs resulting in typically low algal standing crop [45], [46]. The higher rates of primary production and grazing on shallower reefs could magnify the importance of species-specific effects of herbivores.

Increasing emphasis is being placed on the differences among herbivores species in controlling ecological processes on reefs [15], [16], [17], [18]. One important difference is that different diet choices or feeding disturbance rates among herbivores can alter the successional trajectory of the community [37], [38]. These differences in feeding may determine whether algal turfs or upright macroalgae dominate. For example, urchins may inhibit succession and promote algal communities dominated by a low standing crop of early successional, short turfs while herbivorous fishes promote succession to communities with either a larger standing crop of turfs or with increased abundance of macroalgae [2], [47]. In our study, videotapes of free-ranging reef fishes showed that *Scarus* spp. parrotfishes (primarily princess parrotfish and the striped parrotfish, *Scarus iserti*) were responsible for ∼70% of the feeding on cinderblocks, *Acanthurus* spp. surgeonfishes (mostly the ocean surgeonfish) were responsible for ∼25%, and *Sparisoma* spp. parrotfishes (almost exclusively the redband parrotfish) were responsible for only 5% [48]. Thus, ocean surgeonfish and princess parrotfish, which often target algal turfs [16], [27], [29], were rapid and frequent disturbers of early successional substrates in addition to feeding off of the established benthic communities; their feeding kept blocks in early successional stages due to these frequent disturbances (also see ref. 36). When new substrate becomes available (as might occur following coral death due to bleaching or disease), rapid grazing by ocean surgeonfish and princess parrotfish may facilitate dominance by early successional filamentous algae that tolerate grazing or by coralline crusts that resist damage from these herbivores [5], both of these algal states can facilitate coral recovery [39]. This process will be density-dependent however. Below some threshold density of herbivores or above some threshold area of new substrate, reduced grazing on small turfs may allow macrophytes to establish and suppress both the preferred prey of these herbivores and coral settlement, growth, and survivorship [7], [10], [49]. Further, on the established benthic community where initial macroalgal cover was 35–40%, neither surgeonfish-only nor princess parrotfish-only were able to prevent macroalgae from increasing by 130–150% [16]. Redband parrotfish, however, appear to be important for controlling macroalgae in established communities [16] and they (and other *Sparisoma* parrotfishes) may be of primary importance for removing stands of macroalgae. Evidence from the Bahamas supports the idea that *Sparisoma* spp. in particular are important for lowering macroalgal abundance as recovery of *Sparisoma viride* in a marine protected area was associated with declines in upright macroalgae and increases in coral settlement [13], [50]. *Sparisoma* spp. may have little influence on the recruitment of macroalgae in the early stages of succession, instead preferring to feed on established macrophytes [16], [28]. As an example, redband parrotfish had minimal effects on the establishment of the most abundant macroalga, *Dasycladus vermicularis*, in our cages. Adults of *D. vermicularis* were unpalatable to both redband parrotfish and ocean surgeonfish in direct feeding assays, but feeding by surgeonfish suppressed this alga's establishment while feeding by redband parrotfish did not. Thus, ocean surgeonfish likely removed germlings of *D. vermicularis* before they grew large enough to be recognized and avoided. These data emphasize the value of experiments in determining the impacts of consumers on community structure. Had we measured only feeding by these herbivores on a suite of macroalgae, we would have concluded that ocean surgeonfish would not affect the abundance of *D. vermicularis* due to their avoidance of mature thalli. Instead, feeding by ocean surgeonfish prevented the establishment of most macroalgae, even species that are unpalatable when mature. Similar patterns exist on Indo-Pacific coral reef where there are significant interspecific differences among herbivorous fishes in terms of whether they feed on early or late successional algal species [17].

Coastal marine systems often suffer losses of ecosystem function following reductions in biodiversity [51], making it important to understand how individual species and combinations of species [16] function in ecosystems. We show that the different feeding preferences documented across species of herbivorous fishes [16], [27], [28], [29], [30] can translate into substantial differences in the direct and indirect effects that these species have on communities and that these effects will depend on the initial community structure and successional stage of the benthic community. Thus, different herbivore species can play fundamentally different roles in determining the extent of turf and/or upright macroalgae in the algal community [17], and the impact of particular herbivore species on reef communities may be underestimated if they are only evaluated under a limited set of conditions (e.g. low macroalgal abundance). Because these fishes appear to have strong species-specific effects on communities, functional redundancy in these systems may be low with limited overlap in feeding preferences or community impact among herbivores in the same genus [18]. The loss of ecosystem function with the loss of only a few consumers from marine ecosystems [15], [52] suggests that research assessing the complex patterns of functional diversity and redundancy within herbivore guilds will improve our understanding of how declining herbivore diversity may affect reef health.

## Materials and Methods

### Ethics Statement

All procedures were approved by the Institutional Animal Care and Use Committee at the Georgia Institute of Technology.

### Experimental Setup and Maintenance

We tested the roles of herbivore identity and richness on algal succession at Conch Reef (24°57′N/80°27′W) in the Florida Keys, USA. Conch Reef is a fringing reef approximately 8 km southeast of Key Largo, FL. It is within a Special Protection Area within the Florida Keys National Marine Sanctuary where all fishing has been prohibited since 1997. The reef is a spur and groove reef formation that is dominated by upright macroalgae (30–40% cover, mostly *Dictyota* spp. with lesser amounts of *Lobophora variegata*, and *Halimeda* spp.), filamentous turf algae (∼25% cover), and crustose coralline algae (20–25% cover). Live coral cover is 6–7%, with sponges and gorgonians each occupying 5–7% cover.

At a depth of 16–18 m on Conch Reef, we constructed 32 cages of 2 m×2 m×1 m tall from 0.6 cm steel bar supporting PVC-coated, galvanized wire (2.5 cm mesh). Cage frames were affixed to 30 cm galvanized nails hammered into the reef. A 30 cm flange of mesh at the cage base was tightly conformed to the reef and affixed using galvanized fencing nails. Zinc anodes prevented corrosion. For Year 1, we enclosed redband parrotfish (*Sparisoma aurofrenatum*) and ocean surgeonfish (*Acanthurus bahianus*) to create the following treatments: (1) two redband parrotfish, (2) two ocean surgeonfish, (3) one redband parrotfish and one ocean surgeonfish, or (4) no enclosed fishes. Fishes were 14–18 cm standard length for redband parrotfishes and 12–16 cm standard length for ocean surgeonfishes. Fish mass was statistically indistinguishable among treatments, fishes in cages grazed at rates similar to free-ranging conspecifics, and the physical condition (mass/length) of caged fishes at the end of the 10-month experiment was equivalent to uncaged conspecifics from the surrounding reef (see ref. 17). Four cages were blocked spatially (within 3–4 m of each other), and treatments were allocated randomly among each of the four cages. We constructed eight blocks, each containing all four experimental treatments. We did not use cage controls (open-sided cages) in the experiment as all treatments had cages around them and we were not comparing treatments with vs. without cages. Thus, all treatments would have been subject to the same alterations in the physical environment imparted by the cages making controls for caging artifacts unnecessary. However, artifacts from cages built of this mesh are minimal in terms of algal community development [53], water flow [54], or sedimentation in cages [55]. Every 4–6 weeks, we surveyed fishes inside the cages, replaced missing fishes, and scrubbed the cages to remove fouling organisms (see ref. 16 for details of treatment maintenance). The experiment in Year 1 ran from November 2003 until August 2004.

In November 2004, we set up the Year 2 experiment using the same design, except we used redband parrotfish and princess parrotfish (*Scarus taeniopterus*). Fishes were intermediate phase and were 14–19 cm standard length for redband parrotfishes and 15–22 cm standard length for princess parrotfishes. Again, herbivore mass was similar across all treatments. For both years, density and biomass of enclosed herbivorous fishes per area was within the range seen on present-day Caribbean reefs [16], [56]. The experiment ran from November 2004 until July 2005 when surge from Hurricane Dennis destroyed the cages. However, all data presented for Year 2 were collected in June 2005 before the cages were destroyed and while the herbivore treatments were intact.

Cinderblocks (∼10 cm×20 cm×40 cm) were used as substrate for the development of algal communities because they are easily anchored to the reef with large spikes, and are readily colonized by an algal community that is indistinguishable from communities on natural coral skeleton [53]. Each cage contained two cinderblocks, one enriched with nitrogen and phosphorous and one unenriched. We present data only from the unenriched cinderblocks as the nutrient enrichment had minimal effects on algal community structure (D.E.B. and M.E.H. unpub. data). At the end of Year 1, all cinderblocks were brought to the surface and scraped of all algae. The cinderblocks were then soaked in a dilute chlorine bleach solution for ∼30 min, scrubbed with a brush to remove remaining organisms, and then soaked in fresh water. The cinderblocks were then stored dry for 10 weeks before being redeployed for Year 2.

### Quantifying Algal Community Development

Every 11–14 weeks, we sampled the community composition of algae on each cinderblock by identifying the algae under each of 100 points within a 15 cm×30 cm quadrat placed over each cinderblock. We identified algae to the lowest taxonomic level possible in the field, but lumped algae into genera or morphological groups when species-level identification was problematic (e.g. filamentous algae <0.5 cm tall, filamentous algae >0.5 cm tall, crustose coralline algae). Invertebrates were rare and represented less than 3% cover in all treatments. In August 2004, cinderblocks were wrapped individually in plastic bags and brought to the surface where they were lightly scraped with a paint scraper to remove algal biomass (except for crustose coralline algae). Algae were sorted to species or genus and then dried to a constant weight at 60°C. Hurricane Charley passed within 150 km of our field site two weeks before biomass data were gathered so some poorly attached algae such as mats of tall filamentous algae were dislodged via wave action from the hurricane. Thus, these data on biomass primarily represent the mass of upright macroalgae. Data on biomass from Year 2 were not gathered due to the termination of the experiment by Hurricane Dennis.

To evaluate how the herbivore treatments affected algal abundance, we used repeated measures two-factor ANOVA for the cover of different algal functional groups. Additionally, we grouped upright macroalgae, cyanobacteria, and taller algal turf (>0.5 cm) to estimate the overall level of competition from algae because these growth forms are most likely to cause mortality and competitive suppression of corals [10]. We excluded crustose coralline algae and short (<0.5 cm) turfs from this group because we would not expect these low growing forms to affect corals in the size classes that we used in our experiment. Upright macroalgae in Year 2 were present on blocks in only 3 out of 28 cages and were not analyzed due to this low abundance. For algal biomass data, we used two-factor ANOVA on data from the final sampling period. Data were rank or log transformed when necessary to achieve normality and alleviate heterogeneity of variance among the data. In Year 1, we excluded four replicates from the analyses due to persistent loss of fishes from these cages which resulted in *n* = 6 for the redband-only and the mixed-herbivore treatments. In Year 2, consistent fish loss necessitated the removal of four replicates resulting in *n* = 7 for the mixed-herbivore treatment and *n* = 5 for the redband-only treatment. We consistently noted moray eels in these replicates, suggesting an obvious reason for fish loss. However, in the remaining replicates the treatments were intact for approximately equal periods of time for both years [16].

We assessed the effect of herbivore richness on macroalgal abundance using resampling statistics (resampling 10,000 times with replacement) to compare the mixed-herbivore treatment to each single-herbivore treatment [57] (see ref. 16 for similar use of resampling statistics). Significant effects of herbivore richness would occur if algal abundance in the mixed-herbivore treatment was either higher or lower than both single-herbivore treatments. For time series data on percent cover, we used data from the final sampling period to test for herbivore richness effects. We controlled the error rate using the Bonferroni correction, i.e., α = 0.025 for each test. To assess similarity in algal communities across treatments within both years of the experiment, we performed ordination using nonmetric multidimensional scaling (NMDS). For Year 1, we used cover data from the last sampling period in July 2004 and biomass data from August 2004 to build the data matrix. For Year 2, the original data matrix consisted of the cover data for the common algal functional groups in May 2005. We used Sørensen (Bray-Curtis) distance to generate the distance matrix for the analysis.

In Year 1, we performed a feeding assay with *Dasycladus vermicularis*, the most common macroalga, to determine its palatability to redband parrotfish and ocean surgeonfish.

Four individual thalli of *D. vermicularis* (each ∼4 cm tall) were entwined into three-strand ropes. One rope was then placed in each single-herbivore and exclosure cage for 24–30 h (*n* = 5−8 separate cages). Percent removal of each thallus was visually estimated and categorized as 0, 25, 50, 75, or 100% removed as compared to a 4 cm guide. The amount removed from each thallus was averaged for the four thalli per rope within a treatment. Because we could not detect any removal of *D. vermicularis* in the herbivore exclosures, we tested for significant feeding on the alga in the single-herbivore treatments using a Wilcoxon signed-rank test to compare the feeding in each treatment to zero.

### Effects of Treatments on Coral Growth

To evaluate the effect of herbivore treatments on coral growth, one individual each of the massive coral *Porites astreoides* (∼70–80 mm diameter individuals) and the branching coral *Porites porites* (∼80–90 mm branches) were attached to each cinderblock with underwater epoxy at the initiation of Year 1. *P. astreoides* individuals were collected whole from the benthos while branches of *P. porites* were removed from larger colonies. To create a benchmark from which to measure coral growth at the end of the experiment, coral pieces were incubated in clear plastic bags *in situ* for seven hours per day over two days with seawater and alizarin red (∼20 mg/L). During incubation alizarin red is incorporated into the coral skeleton and produces a band of color from which to measure growth. At the conclusion of the experiment, remaining corals were collected and sectioned with a diamond saw. *P. porites* was sectioned down the growth axis (i.e. from base to tip of the branch), and we measured linear extension of the skeleton (i.e. increase in branch length) as the length of coral skeleton distal to the alizarin red band. *P. astreoides* was sectioned down the vertical axis of the midpoint of each colony, and we measured the increase in thickness of the skeleton at the apex of the skeleton. Corals were not transplanted to cinderblocks in Year 2. Growth for both coral species was assessed by performing a two-factor ANOVA on log-transformed data. We tested for effects of herbivore richness using resampling statistics as described. Because overgrowth by algae often leads to suppressed growth in corals [8], [58], [59], we used least squares linear regression to test the relationship between total algal cover and coral growth for both *P. porites* and *P. astreoides*. Total algal cover was defined as the sum of upright macroalgal cover and tall filamentous turf algae as these groups are most likely to affect coral growth [10].

## Supporting Information

Table S1 Year 1 results from repeated measures, two-factor ANOVA of percent cover data. Significant effects are highlighted in bold.
(0.08 MB PDF)

Table S2 Year 2 results from repeated measures, two-factor ANOVA of percent cover data. Significant effects are highlighted in bold.
(0.07 MB PDF)
