# Supplementary material for: Impact of Herbivore Identity on Algal Succession and Coral Growth on a Caribbean Reef
Source: PLoS One. 2010 Jan 29;5(1):e8963. doi: 10.1371/journal.pone.0008963 (PMC2813280; doi:10.1371/journal.pone.0008963)
Supplement: Table S1 — Year 1 results from repeated measures, two-factor ANOVA of percent cover data. Significant effects are highlighted in bold. (0.08 MB PDF) [file pone.0008963.s001.pdf]

Table S1 - Year 1 results from repeated measures, two-factor ANOVA of percent cover data. Significant effects are highlighted in bold.

		**Upright macroalgae**		**Cyanobacteria**		**Algal turf (>0.5cm)**	
**Source**	***df***	***F***	***P***	***F***	***P***	***F***	***P***
Between subjects (mean effect over time)							
Redband	1,24	0.01	0.916	0.01	0.916	0.04	0.841
Ocean	1,24	51.11	**<0.001**	0.19	0.660	12.71	**0.002**
Redband X Ocean	1,24	4.07	0.055	0.08	0.782	0.23	0.634
Within subjects (change in effect over time)							
Time	2,23	9.95	**<0.001**	3.51	**0.047**	2.03	0.154
Time X Redband	2,23	5.24	**0.013**	0.35	0.703	2.20	0.134
Time X Ocean	2,23	7.44	**0.003**	0.12	0.887	1.79	0.189
Time X Redband X Ocean	2,23	0.01	0.995	0.79	0.465	0.69	0.510
		Dasycladus		***Codium***		***Dictyota***	
**Source**	***df***	***F***	***P***	***F***	***P***	***F***	***P***
Between subjects (mean effect over time)							
Redband	1,24	0.29	0.597	0.95	0.339	4.77	**0.039**
Ocean	1,24	15.08	**<0.001**	3.23	0.085	4.63	**0.042**
Redband X Ocean	1,24	2.03	0.167	0.84	0.369	3.99	0.057
Within subjects (change in effect over time)							
Time	2,23	13.78	**<0.001**	3.63	**0.043**	4.14	**0.029**
Time X Redband	2,23	1.22	0.313	1.62	0.219	0.73	0.495
Time X Ocean	2,23	8.48	**0.002**	2.12	0.143	1.22	0.315
Time X Redband X Ocean	2,23	0.33	0.720	1.39	0.268	1.16	0.332
		**Macro, cyano, turf**		**Algal turf (<0.5cm)**		**Crustose corallines**	
**Source**	***df***	***F***	***P***	***F***	***P***	***F***	***P***
Between subjects (mean effect over time)							
Redband	1,24	3.48	0.075	2.87	0.103	1.83	0.189
Ocean	1,24	153.05	**<0.001**	48.78	**<0.001**	3.40	0.078
Redband X Ocean	1,24	4.67	**0.041**	3.48	0.074	<0.01	0.994
Within subjects (change in effect over time)							
Time	2,23	12.70	**<0.001**	9.04	**0.001**	3.28	0.055
Time X Redband	2,23	6.04	**0.008**	1.40	0.266	1.50	0.243
Time X Ocean	2,23	0.37	0.695	4.02	**0.032**	1.75	0.195
Time X Redband X Ocean	2,23	0.52	0.600	1.83	0.182	0.08	0.922
