# Supplementary material for: Impact of Herbivore Identity on Algal Succession and Coral Growth on a Caribbean Reef
Source: PLoS One. 2010 Jan 29;5(1):e8963. doi: 10.1371/journal.pone.0008963 (PMC2813280; doi:10.1371/journal.pone.0008963)
Supplement: Table S2 — Year 2 results from repeated measures, two-factor ANOVA of percent cover data. Significant effects are highlighted in bold. (0.07 MB PDF) [file pone.0008963.s002.pdf]

Table S2 - Year 2 results from repeated measures, two-factor ANOVA of percent cover data. Significant effects are highlighted in bold.

		**Upright macroalgae**		**Cyanobacteria**		**Algal turf (>0.5cm)**	
**Source**	***df***	***F***	***P***	***F***	***P***	***F***	***P***
Between subjects (mean effect over time)							
Redband	1,24	NA	NA	3.39	0.078	4.29	**0.049**
Princess	1,24	NA	NA	0.26	0.617	27.93	**0.010**
Redband X Princess	1,24	NA	NA	0.30	0.585	6.25	**0.020**
Within subjects (change in effect over time)							
Time	2,23	NA	NA	3.72	0.066	6.46	**0.018**
Time X Redband	2,23	NA	NA	2.12	0.158	1.77	0.196
Time X Princess	2,23	NA	NA	0.17	0.676	0.02	0.968
Time X Redband X Princess	2,23	NA	NA	2.05	0.165	2.40	0.134
		**Macro, cyano, turf**		**Algal turf (<0.5cm)**		**Crustose corallines**	
**Source**	***df***	***F***	***P***	***F***	***P***	***F***	***P***
Between subjects (mean effect over time)							
Redband	1,24	3.05	0.093	1.18	0.288	0.72	0.431
Princess	1,24	10.57	**0.003**	32.81	**<0.001**	37.41	**<0.001**
Redband X Princess	1,24	6.91	**0.015**	16.48	**<0.001**	2.69	0.114
Within subjects (change in effect over time)							
Time	2,23	1.93	0.152	4.64	**0.041**	2.76	0.109
Time X Redband	2,23	2.48	0.128	0.03	0.863	2.03	0.167
Time X Princess	2,23	0.64	0.429	2.92	0.101	1.66	0.209
Time X Redband X Princess	2,23	0.24	0.626	3.13	0.090	2.65	0.116
